# Discrimination of Pharmaceutical Tablets Based on the Analysis of Solid-State Structures of Ingredients Using Terahertz Transmission Spectroscopy with the Injection-Seeded Parametric Generation Technique

**DOI:** 10.1021/acsomega.1c04121

**Published:** 2021-10-01

**Authors:** Kei Shimura, Mizuki Mohara, Kenji Aiko, Tomoaki Sakamoto, Touya Ono

**Affiliations:** † Hitachi High-Tech Corporation, Shinko-cho, Hitachinaka 312-8504, Japan; ‡ National Institute of Health Sciences, Tonomachi, Kawasaki 210-9501, Japan

## Abstract

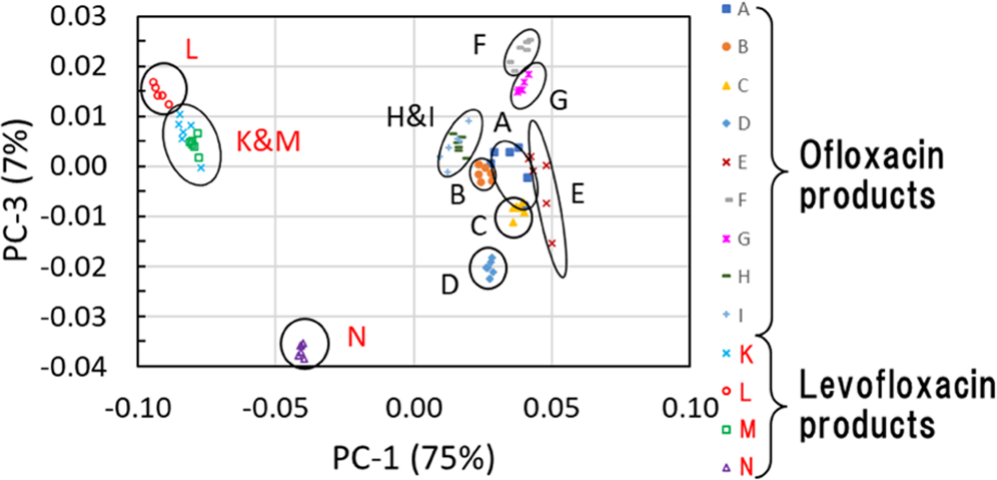

A frequency-domain terahertz (THz) spectrometer that uses a tunable source, called an injection-seeded THz parametric generator, was applied to the analysis of solid-state structures of ingredients in pharmaceutical tablets, and its performance on discriminating pharmaceutical products was evaluated. The spectrometer has a dynamic range of 70 dB at 2 THz and is suitable for analyzing materials such as pharmaceutical ingredients that often have characteristic absorption peaks between 0.5 and 2.5 THz. Nine ofloxacin (racemate) and four levofloxacin (levorotatory enantiomer) tablet products commercially available in Japan were used as samples. They contain 8–12 additives in addition to the API. The sample tablets were filed down to a thickness of 1.2 mm (ofloxacin tablets) and 1.6 mm (levofloxacin tablets) to obtain transmission spectra over the wide spectral range of 0.8–2.1 THz. The absorption spectra obtained from the spectrometer were preprocessed by the second derivative; then, principal component analysis (PCA) was conducted on the results. Next, quadratic discriminant analysis (DA) was conducted on the scores of the three PCA components. The accuracy of the DA for all 13 products was 96.1%. In addition to the difference in crystal forms of the active ingredient, the small differences in the formulation were clearly discriminated using the THz absorption spectra. The spectrometer combined with data analysis shows potential for applications such as identifying pharmaceutical tablets, monitoring the stability of production processes, evaluating the stability of formulations during storage, and detecting counterfeit drugs on the market.

## 1 Introduction

The analysis of crystal properties, such as crystal forms and crystallinity, of active pharmaceutical ingredients (APIs) in formulation tablets is important for quality control of pharmaceutical products.^1^ The crystal form selected for each product depends on the brand, and its crystallinity could change in the production process. Furthermore, the dissolution rate of APIs depends on their crystal forms; amorphous forms generally have a higher dissolution rate than crystal forms. Various crystal forms including crystal of salts, solvates, and cocrystals have been investigated to improve the dissolution rate of poorly water-soluble APIs. In addition, the stability of the products could depend on the crystal properties since amorphous and metastable crystal forms tend to transform to more stable forms over time.

Various analytical techniques, such as thermal analysis, X-ray diffraction (XRD), infrared (IR) and near-infrared (NIR) spectroscopy, and Raman spectroscopy, have been used to study crystal forms and crystallinity in pharmaceutical samples. Although XRD and thermal analysis are the benchmarks for analyzing crystal forms, they are mainly used for the analysis of pure substances. IR, NIR, and Raman spectroscopy with chemometrics are expected as process analytical technologies.^2,3^ However, most research has been based on reflection measurements, and the analysis inside formulation tablets was left for future study.

Terahertz (THz) transmission spectroscopy is expected as an analytical method for the crystal properties of the APIs in formulation tablets^4^ since the vibration of lattice phonon modes originating in a crystal structure can be measured and major pharmaceutical additives are transparent or semitransparent in the THz frequency region. Many APIs, their crystal polymorphs, and their hydrates and cocrystals have characteristic absorption peaks in this frequency region.^5−9^ In addition, quantitative analysis of the crystal forms of pure APIs through THz transmission measurements has been reported.^10^ Quantitative analysis of ternary mixtures of pharmaceutical additives has also been achieved using chemometrics.^11^ It is known that enantiomers and racemic compounds can be analyzed quantitatively.^12,13^

However, its application to formulation tablets is limited so far because it is not easy to obtain absorption peaks in a frequency region higher than 1.5 THz with nondiluted formulation tablets using commonly used THz time-domain spectroscopy (TDS) systems.^14−19^ In THz-TDS systems, the dynamic range of measurements decreases as frequency increases.^20^ On the other hand, scattering in sample tablets increases as the thickness of the sample increases and as the frequency increases. Therefore, in transmission measurements, the upper limit of the spectral range is limited by the thickness in addition to the properties of the ingredients. Hisazumi et al. reported that a practical spectral range for their 1.5 mm thick mock formulation tablets was between 0.5 and 1.6 THz.^19^ Taday et al. showed spectra of nondiluted mock formulation tablets up to 3 THz, but the thickness of the tablets was 0.5 mm.^15^ Although clear spectra up to 3 THz were also obtained through measurements on 1 mm thick samples made by crushing commercial formulation tablets, diluting them with polyethylene, and compressing them again to tablets,^14^ this preparation process may change the crystal forms of the APIs. Much simpler and less destructive preparations are necessary for this application.

We have developed a new frequency-domain (FD) THz spectrometer that uses a high-peak-power tunable THz source called an injection-seeded THz parametric generator (is-TPG).^21−24^ We demonstrated that clear absorption peaks can be obtained in the spectral range between 1.0 and 2.4 THz for thick (*t* = 4.1 mm) nondiluted formulation tablets through transmission measurements, and the crystal forms of an API in low-dose (<5 wt %) formulation tablets can be identified with this spectrometer.^25^ We also showed that hydration and dehydration of an API in formulation tablets can be clearly observed with this spectrometer.^26^ Therefore, this spectrometer could be used to analyze the crystal properties of APIs inside formulation tablets or inspect the stability of the APIʼs solid-state structure during storage.

In this study, we applied our spectrometer to the discrimination of commercial formulation tablets and evaluated its performance on discriminating the difference in the crystal properties of an API and small differences in formulation. We selected nine ofloxacin tablet products and four levofloxacin tablet products. Ofloxacin (C_18_H_20_FN_3_O_4_) is a quinolone antibiotic useful for the treatment of bacterial infections. It is a racemic mixture of levofloxacin (levorotatory enantiomer) and dextrofloxacin (dextrorotatory enantiomer). Levofloxacin is biologically active but dextrofloxacin is inactive. Therefore, we could evaluate whether a crystal of racemate and that of levorotatory enantiomer could be differentiated even in formulation tablets. We obtained the THz absorption spectra of the tablets through transmission measurements using our THz spectrometer. Due to the strong scattering inside the tablets, we needed to reduce their thickness to obtain reasonable spectral range, but we could keep it greater than 1 mm. We applied the second derivative on the spectra and conducted principal component analysis (PCA) on the results.^27^ We then attempted to discriminate those products by conducting quadratic discriminant analysis (DA) on the scores of the three PCA components.

## 2 Experimental Section

### 2.1 Sample and Preparation

To confirm the characteristic absorption peaks of the APIs, pure ofloxacin and levofloxacin hemihydrate were purchased from Fujifilm Wako Pure Chemical Corporation, and THz absorption spectra were measured. Ofloxacin powder was weighed and pressed into a 10 mm diameter tablet at a pressure of 78.5 kN. Its thickness was 1.0 mm. Levofloxacin hemihydrate powder was weighed and mixed with 10 wt % of polyethylene and pressed into a 10 mm diameter tablet at a pressure of 78.5 kN. Its thickness was 0.84 mm.

Ofloxacin and levofloxacin formulation tablets approved by Japanese Pharmacopoeia were purchased from distributors in Japan. Nine brands for ofloxacin (products A to I) and four brands for levofloxacin (products K to N), including the original drug and its generics, were obtained. The formulations of these tablets are shown in Tables 1 (ofloxacin) and 2 (levofloxacin). The ofloxacin tablets contain 100 mg (50 wt %) of ofloxacin, and the levofloxacin tablets contain 250 mg (75 wt %) of levofloxacin as its hemihydrate form. Six additives are commonly used in ofloxacin products, and the use of the rest of the additives depends on the product. The major difference in terms of THz spectroscopy is the use of lactose hydrate since it shows absorption peaks in the THz region.^28^ Eight additives are commonly used in levofloxacin products, and the use of the remaining four additives depends on the product. This small difference in formulation could make discrimination of levofloxacin products difficult in addition to the high content rate of the API. Six tablets for each product were used as samples. They were filed down to a thickness of 1.2 mm (ofloxacin tablets) and 1.6 mm (levofloxacin tablets) to obtain transmission spectra over the wide spectral range of 0.8–2.1 THz. By avoiding pulverizing the tablets, we could analyze the APIs and additives as they truly are in the product tablets.

**Table 1:** Formulations of Ofloxacin Tablet Products

ingredient	product A	product B	product C	product D	product E	product F	product G	product H	product I
ofloxacin	100 mg	100 mg	100 mg	100 mg	100 mg	100 mg	100 mg	100 mg	100 mg
cornstarch	v	v	v	v	v	v	v	v	v
hypromellose (HPMC)	v	v	v	v	v	v	v	v	v
hydroxypropyl cellulose (HPC)	v	v	v	v	v	v	v	v	v
macrogol 6000 or macrogol	v	v	v	v	v	v	v	v	v
magnesium stearate	v	v	v	v	v	v	v	v	v
titanium oxide	v	v	v	v	v	v	v	v	v
lactose hydrate	v	v	v	v	v	v	v		
sodium starch glycolate							v		
partially pregelatinized starch								v	v
carmellose	v	v							
carmellose calcium			v						
low-substituted hydroxypropyl cellulose (L-HPC)					v	v			
crystalline cellulose				v	v		v	v	
cellulose						v			v
talc	v	v	v				v		
dimethylpolysiloxane	v								
silicon dioxide	v								
carnauba wax	v	v	v			v	v	v	v
polysorbate80							v		
crospovidone								v	v

**Table 2:** Formulations of Levofloxacin Tablet Products

ingredient	product K	product L	product M	product N
levofloxacin	250 mg	250 mg	250 mg	250 mg
crystalline cellulose	v	v	v	v
carmellose	v	v	v	v
hydroxypropyl cellulose (HPC)	v	v	v	v
hypromellose (HPMC)	v	v	v	v
titanium oxide	v	v	v	v
talc	v	v	v	v
macrogol 6000 or macrogol	v	v	v	v
iron(III) oxide monohydrate, yellow	v	v	v	v
carnauba wax	v		v	v
silicon dioxide			v	
sodium stearyl fumarate	v	v		v
magnesium stearate			v	

### 2.2 Acquisition of THz Spectra and Data Processing

**Figure 1:**
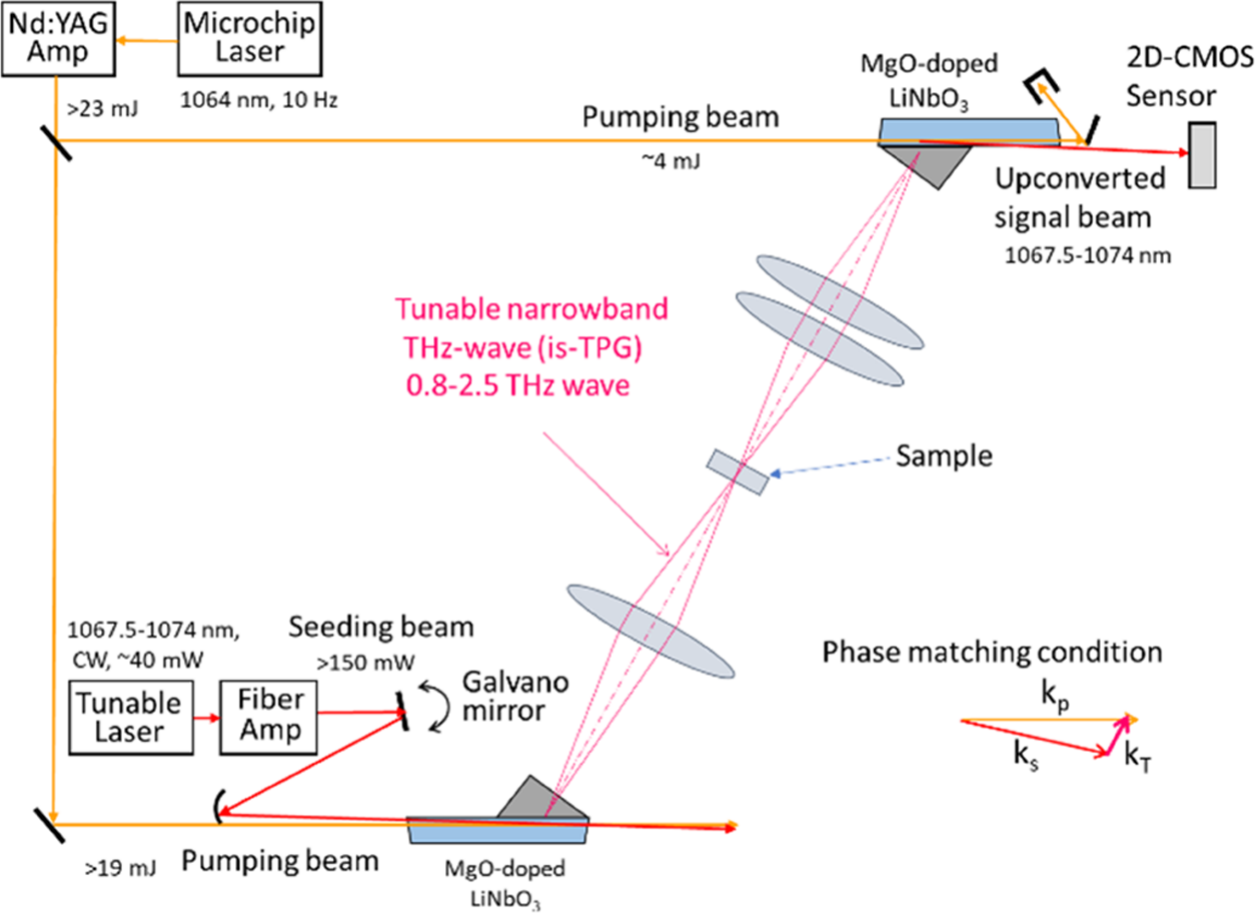
Configuration of optics used in our FD THz spectrometer with is-TPG THz source.

Our FD THz spectrometer^21−24^ that uses a tunable is-TPG THz source was used for obtaining the THz absorption spectra of the sample tablets through transmission measurements. The configuration of the optics is shown in Figure 1. A high-peak-power THz wave was generated by introducing a pumping laser beam and a seeding laser beam to a nonlinear crystal. An output beam from a microchip laser amplified using a neodymium-doped yttrium aluminum garnet (Nd:YAG) amplifier was used as the pumping beam and an output beam from a tunable laser amplified using a fiber amplifier was used as the seeding beam. A magnesium-oxide-doped lithium niobate (MgO-doped LiNbO_3_) crystal was used as the nonlinear crystal. The frequency of the THz wave was scanned from 0.8 to 2.5 THz by changing the wavelength of the seeding laser from 1067.5 to 1074.0 nm. The generated THz wave was focused on a sample tablet in a chamber in which the relative humidity was controlled to less than 2%. The transmitted THz wave was upconverted to near-infrared light using another nonlinear crystal and detected using a two-dimensional-array CMOS sensor at room temperature. The frequency sampling period was set to 0.01 THz. The maximum dynamic range was 70 dB at 2 THz.

The absorption spectra obtained from the spectrometer were preprocessed by the second derivative with a Savitzky–Golay filter (9 points) to suppress baseline variation over samples. The results were then mean-centered, and PCA was conducted on them to reduce the data dimensions before applying DA. DA was conducted on the scores of the three PCA components, and a confusion matrix was obtained. To discriminate the 13 products in the three-dimensional (3D) principal component space, quadratic DA,^29^ in which a covariance matrix is assumed for each product, was conducted. All calculations and modeling were carried out in Unscrambler X Ver.10.5 software (Camo Analytics, Norway).

## 3 Results and Discussion

### 3.1 Spectra of Pure Materials

**Figure 2:**
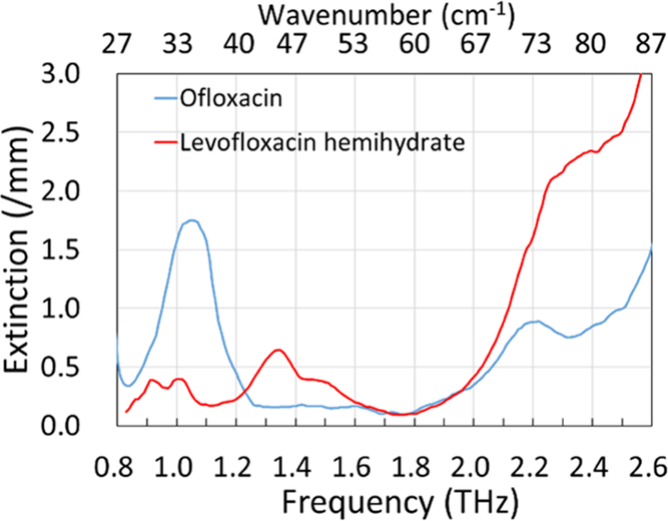
THz absorption spectra of ofloxacin (racemate) (blue) and levofloxacin hemihydrate (levorotatory enantiomer) (red).

The absorption spectra of pure ofloxacin and levofloxacin hemihydrate are shown in Figure 2. The vertical axis shows the absorbance obtained from the spectrometer, but we use “extinction” instead of “absorbance” in this paper because strong attenuation by scattering inside a sample tablet is sometimes observed in THz spectroscopy. We also normalized the extinction value by the thickness of the samples measured in millimeters to reduce the effect of thickness variation. The horizontal axis shows frequency in THz, and we also show the scale in wavenumber at the top for better understanding. The absorption peaks of ofloxacin clearly appeared at 1.05 and 2.22 THz, and those of levofloxacin hemihydrate appeared at 0.95, 1.05, 1.35, and 1.54 THz. We confirmed that these APIs could be identified with these absorption peaks.

### 3.2 Spectra of Formulation Tablets

**Figure 3:**
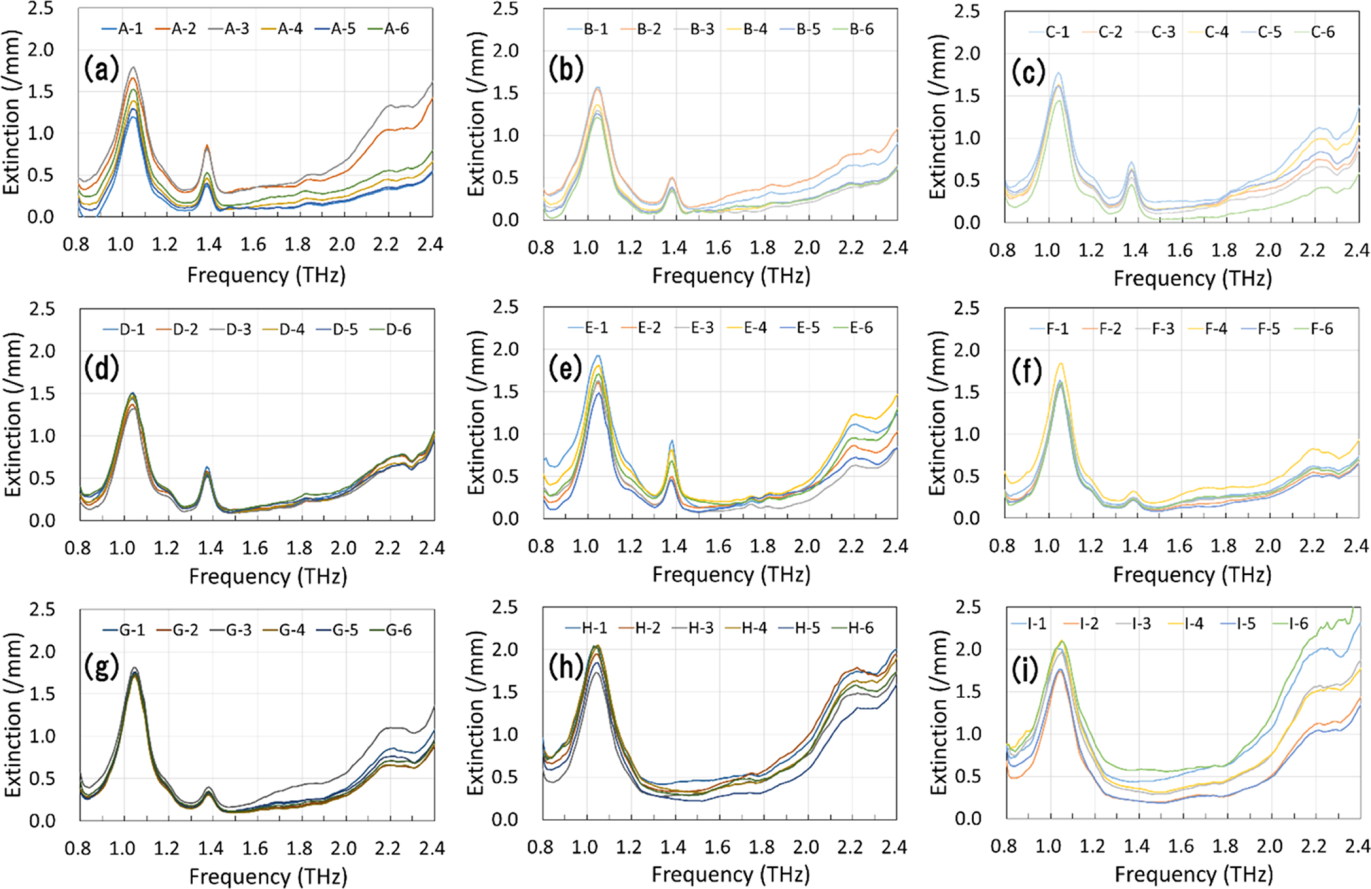
THz absorption spectra of nine ofloxacin tablet products: (a) product A, (b) product B, (c) product C, (d) product D, (e) product E, (f) product F, (g) product G, (h) product H, and (i) product I. Six tablets per product were used as samples.

**Figure 4:**
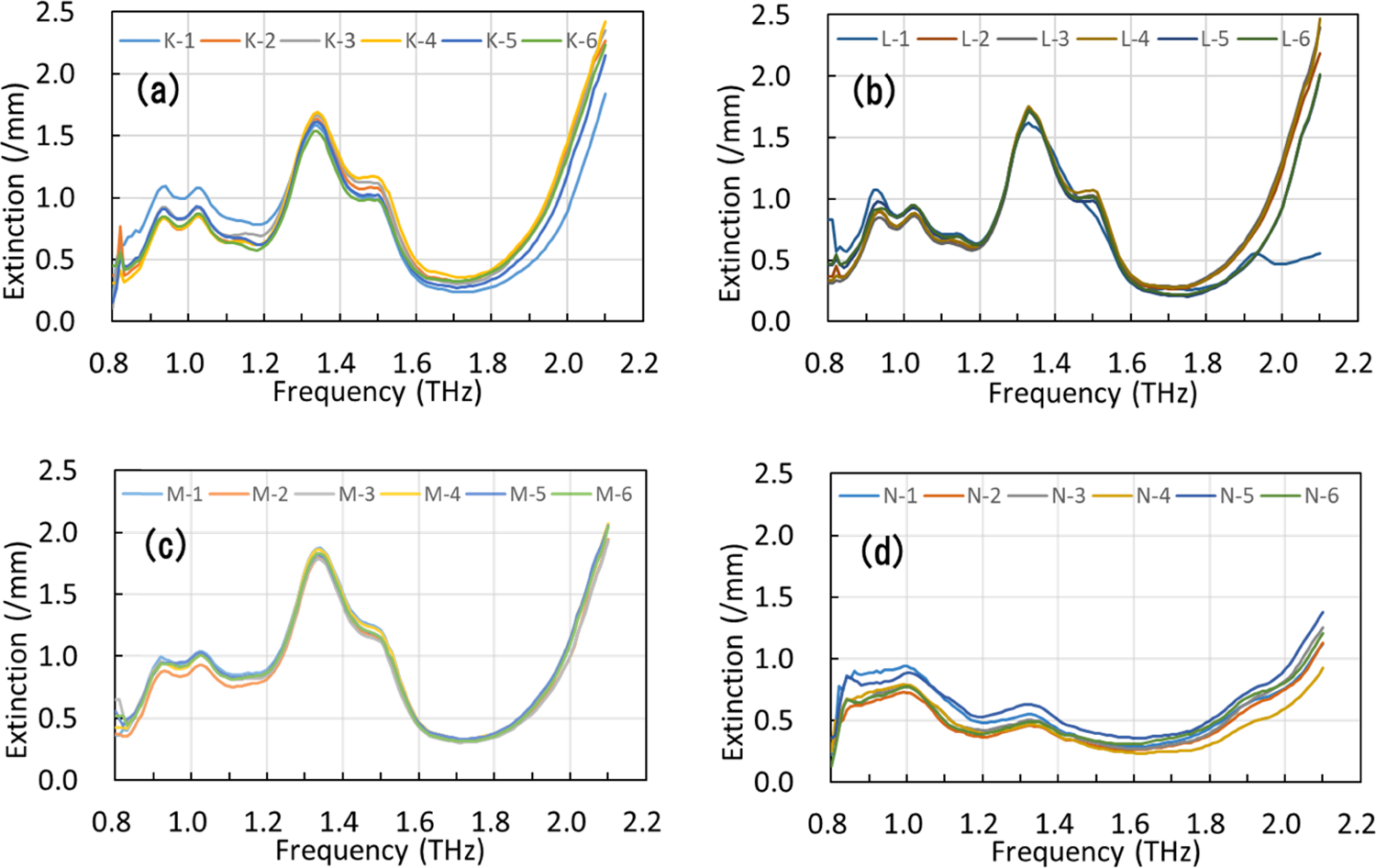
THz absorption spectra of four levofloxacin tablet products: (a) product K, (b) product L, (c) product M, and (d) product N. Six tablets per product were used as samples.

The absorption spectra of the formulation tablets are shown in Figures 3 and 4. Figure 3a–i shows the spectra of ofloxacin tablets. In each figure, the spectra of six sample tablets of the product are shown, i.e., A-1 to A-6. The characteristic peaks of ofloxacin (1.05 and 2.22 THz) were observed in all spectra as expected. The characteristic peaks of lactose monohydrate^28^ (1.20, 1.38, and 1.82 THz) were also observed, as shown in Figure 3a–g. This agrees well with their formulations shown in Table 1. Large baseline variation over six samples from the same product was observed in the spectra of products except products D, F, and G. This may be caused by difference in average particle size, its variation, or its uniformity in pressed tablets. It could show difference in granulation methods used in the production process of these products. However, the variation in the baseline observed in Figure 3 was mostly among samples in the same product and not among products. Therefore, we tried to suppress the variation by preprocessing before discriminant analysis. Figure 4a–d shows the spectra of the levofloxacin tablets. The characteristic peaks of levofloxacin hemihydrate (0.95, 1.05, 1.35, and 1.50 THz) were clear in Figure 4a–c and not clear in Figure 4d. This indicates that the crystal form of levofloxacin in product N was different from that in other products since the amount of levofloxacin in product N was confirmed through HPLC measurement. The powder X-ray diffraction (PXRD) result of the pulverized product N tablet showed the mixture of diffraction peaks of levofloxacin hemihydrate and levofloxacin monohydrate (as shown in the Supporting Information), but further analysis is necessary to identify the crystal form in the product since it could be changed by pulverization. One spectrum obtained from one of the tablets of product L was not used for further analysis since the data over 1.9 THz showed different behavior from those over other spectra.

### 3.3 Principal Component Analysis

**Figure 5:**
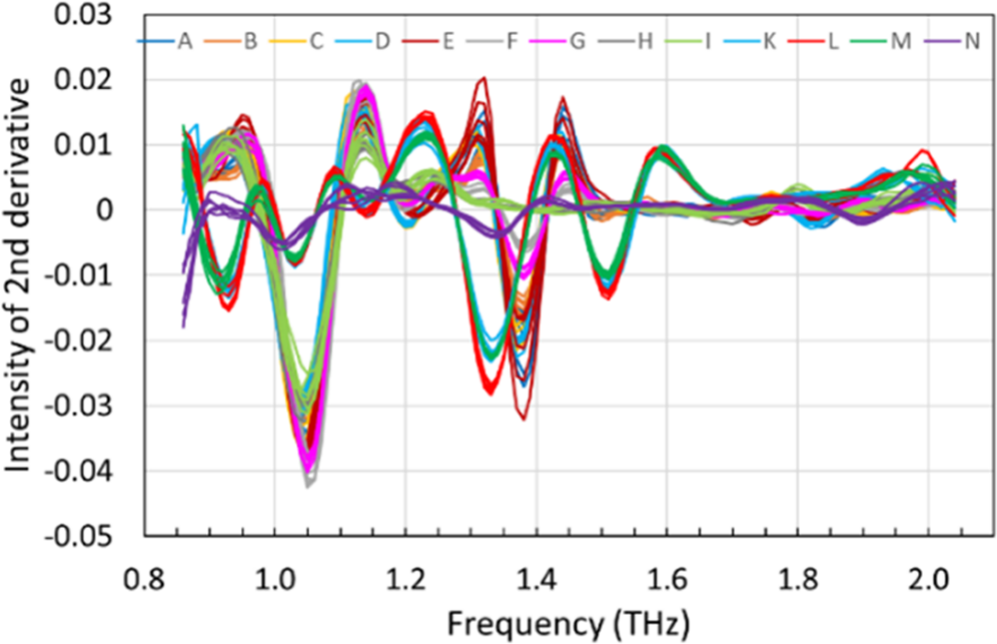
Second derivative of the THz spectra of 13 products: nine ofloxacin tablet products (products A–I) and four levofloxacin tablet products (products K–N).

The spectra obtained from our spectrometer were preprocessed by the second derivative with a nine-point Savitzky–Golay filter. The results are shown in Figure 5. The characteristic absorption peaks of the APIs and lactose were clear, and the dependence of the spectral features on each product became much more apparent by preprocessing. The results were then mean-centered, and PCA was conducted to reduce the data dimensions. The PCA results indicate that three components were necessary to explain the variance in the spectra, and the ratio of the explained variance for the first, second, and third principal components were 75, 11, and 7%, respectively.

**Figure 6:**
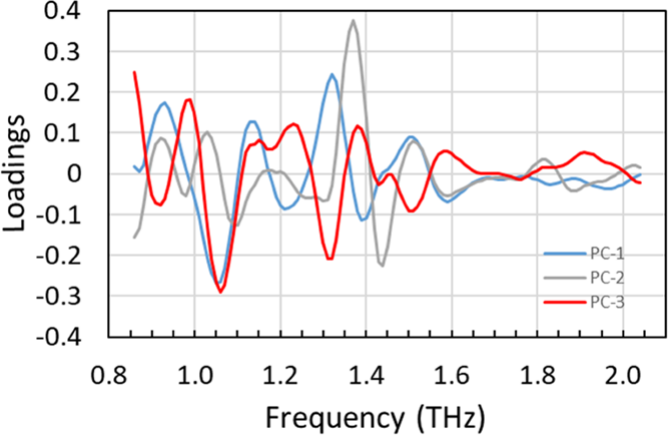
PCA loadings of first (blue), second (gray), and third (red) components.

The PCA loading plots are shown in Figure 6. The loading of the first principal component (PC-1) (blue) showed the strongest negative peak at 1.05 THz, which corresponds to the absorption of ofloxacin, and weak negative peaks at 1.21 and 1.39 THz, which correspond to the absorption of lactose monohydrate. It also showed positive peaks at 0.93, 1.32, and 1.51 THz, which correspond to the absorption of levofloxacin. Therefore, this component was expected to distinguish the ofloxacin product group from the levofloxacin product group. The loading of the second principal component (PC-2) (gray) showed the strongest positive peak at 1.37 THz, which corresponds to the strongest absorption of lactose, and weak positive peaks at 0.92, 1.03, and 1.51 THz, which correspond to the absorption of levofloxacin. Therefore, this would be the component corresponding to the amount and/or the crystallinity of lactose in the ofloxacin product group and that corresponding to the crystallinity of levofloxacin in the levofloxacin product group. The loading of the third principal component (PC-3) (red) showed the strongest negative peak at 1.06 THz, which corresponds to the strongest absorption of ofloxacin, and the second negative peak at 1.31 THz, which was close to the absorption peak of levofloxacin. Therefore, this would be the component corresponding to the crystallinity of ofloxacin in the ofloxacin product group and that corresponding to the crystallinity of levofloxacin in the levofloxacin product group. These results indicate that the PCA was successful, and the sample tablets can be discriminated on the basis of the difference in crystal forms of the APIs, crystallinity of the APIs and additives, and difference in formulations.

**Figure 7:**
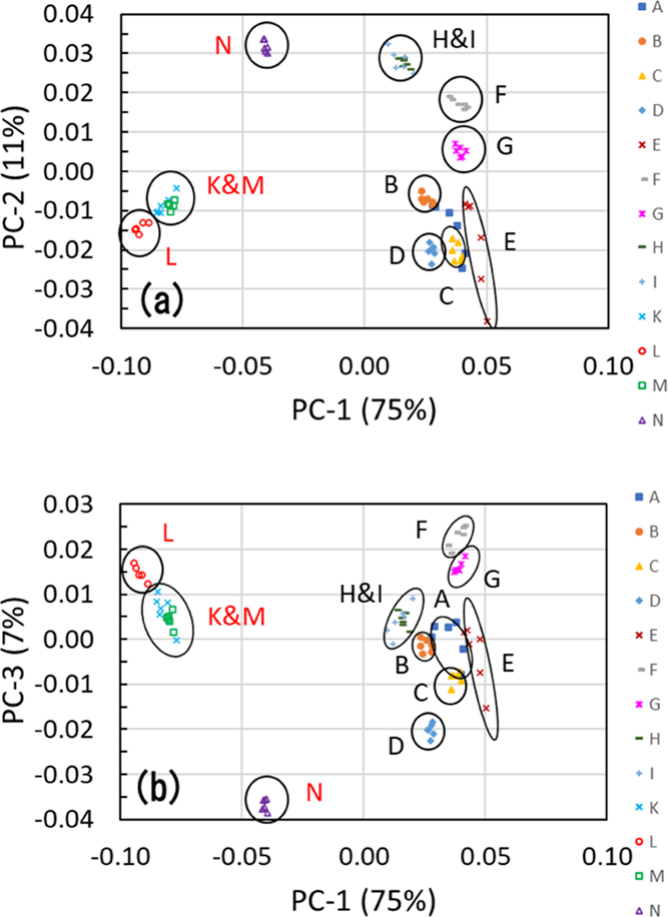
PCA score plots: (a) PC-1 vs PC-2, (b) PC-1 vs PC-3 of THz absorption spectra of nine ofloxacin tablet products (products A–I) and four levofloxacin tablet products (products K–N) preprocessed by the second derivative.

The PCA score plots are shown in Figure 7. Figure 7a,b shows the score plots on PC-1 vs PC-2 plane and PC-1 vs PC-3 plane, respectively. Combining these plots, their 3D distribution can be understood. Figure 7a shows that the ofloxacin and levofloxacin products, or the difference in crystal forms of the APIs, could be clearly distinguished with PC-1. Figure 7a also shows that those products were separated roughly in 10 groups with PC-1 and PC-2. Figure 7b shows that products A and C in the ofloxacin product group could be separated by adding PC-3. These PCA results indicate that these products were discriminated into 11 groups (8 groups for the ofloxacin products and 3 groups for the levofloxacin products) using three components.

### 3.4 Discriminant Analysis

**Figure 8:**
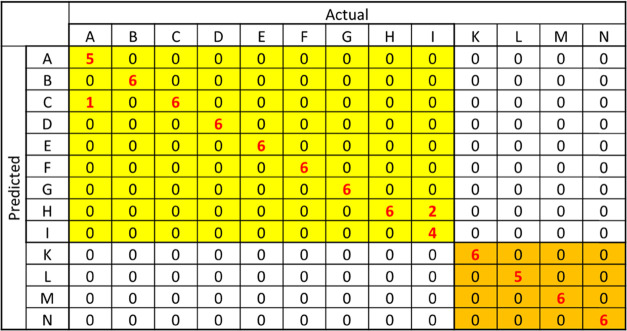
Confusion matrix obtained from quadratic DA of three PCA components of THz absorption spectra of nine ofloxacin tablet products (products A–I) and four levofloxacin tablet products (products K–N).

Quadratic DA was conducted on the scores of the three PCA components and a confusion matrix was obtained. The results are shown in Figure 8. The ofloxacin products and the levofloxacin products were accurately discriminated, and in addition, most of the products were discriminated successfully. Only three tablets were not discriminated correctly. One tablet in product A was predicted as product C, since products A and C had a similar formulation, as shown in Table 1, and were located closely in the score plots as shown in Figure 7. Two tablets in product I were predicted as product H. This result was reasonable because they had almost the same formulation and their score plots overlap. The accuracy of the DA was 96.1%. The small differences in the formulation of commercial tablets were clearly distinguished using the THz absorption spectra obtained from our spectrometer.

## 4 Conclusions

We have applied an FD THz spectrometer that uses a tunable THz source to the discrimination of nine ofloxacin tablet products and four levofloxacin tablet products. In addition to the difference in crystal forms of the APIs, the small differences in the formulation or production process of commercial tablets were clearly distinguished using the THz absorption spectra obtained from our spectrometer. The spectrometer combined with data analysis shows potential for applications such as identifying pharmaceutical tablets, monitoring the stability of production processes, evaluating the stability of formulations during storage, and detecting the counterfeit drugs on the market.

## Supporting Information Available

PXRD pattern of pulverized product N tablet (PDF)
