# Supplementary material for: Discrimination of Pharmaceutical Tablets Based on the Analysis of Solid-State Structures of Ingredients Using Terahertz Transmission Spectroscopy with the Injection-Seeded Parametric Generation Technique
Source: ACS Omega. 2021 Oct 1;6(40):26707–14. doi: 10.1021/acsomega.1c04121 (PMC8515814; doi:10.1021/acsomega.1c04121)
Supplement: Supplementary file 1 — ao1c04121_si_001.pdf [file ao1c04121_si_001.pdf]

Discrimination of Pharmaceutical Tablets Based on the Analysis of Solid-State Structures of Ingredients Using Terahertz Transmission Spectroscopy with the Injection-Seeded Parametric Generation Technique

Kei Shimura[*,1)], Mizuki Mohara[1)], Kenji Aiko[1)], Tomoaki Sakamoto[2)] and Touya Ono[1)]

1) Hitachi High-Tech Corporation, Shinko-cho, Hitachinaka 312-8504, Japan

2) National Institute of Health Sciences, Tonomachi, Kawasaki 210-9501, Japan

* Email:kei.shimura.dh@hitachi-hightech.com

SUPPORTING INFORMATION

Figure S1. (a) PXRD pattern of pulverized Product N tablet and peak positions of (b) levofloxacin hemihydrate (PDF 00-058-1612)[1] and (c) levofloxacin monohydrate (PDF 00-059-0902)[1]

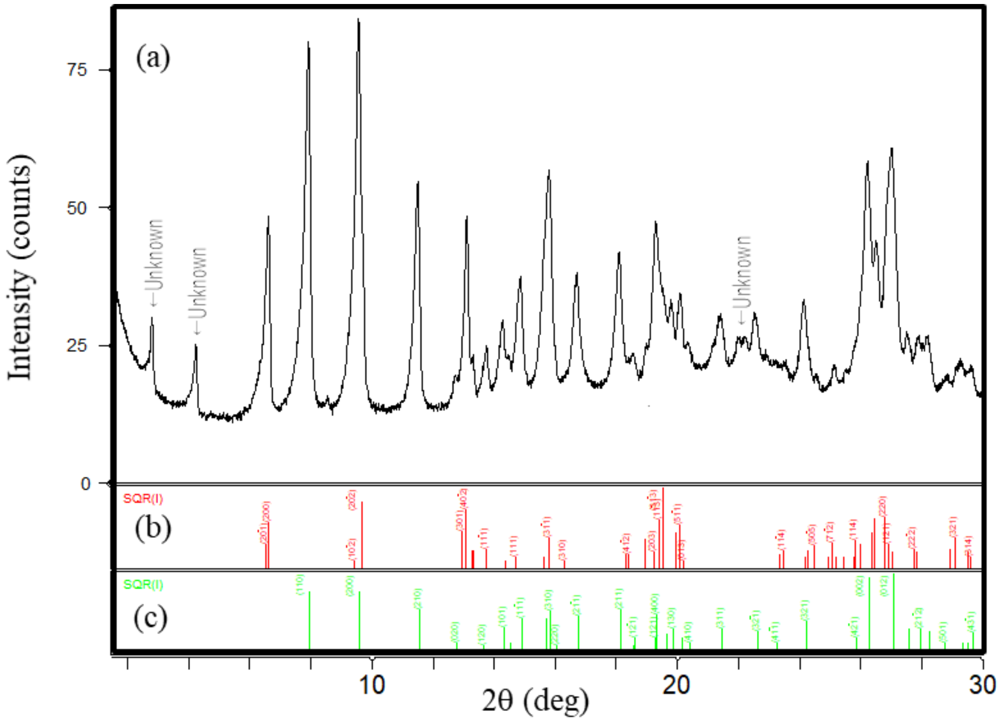

Figure S1. (a) PXRD pattern of pulverized Product N tablet and peak positions of (b) levofloxacin hemihydrate (PDF 00-058-1612)[1] and (c) levofloxacin monohydrate (PDF 00-059-0902)[1]

(1) Gates-Rector, S.; Blanton, T. The Powder Diffraction File: A Quality Materials Characterization Database. Powder Diffr. **2019**, 34 (4), 352–360. https://doi.org/10.1017/S0885715619000812.
